# Integrative study of chicken lung transcriptome to understand the host immune response during Newcastle disease virus challenge

**DOI:** 10.3389/fcimb.2024.1368887

**Published:** 2024-09-03

**Authors:** Venkata Krishna Vanamamalai, E. Priyanka, T. R. Kannaki, Shailesh Sharma

**Affiliations:** ^1^ Bioinformatics Laboratory, DBT-National Institute of Animal Biotechnology (NIAB), Hyderabad, Telangana, India; ^2^ Graduate Studies, Regional Centre for Biotechnology (RCB), Faridabad, Haryana, India; ^3^ Laboratory of Avian Health and Pathology, ICAR-Directorate of Poultry Research, Hyderabad, Telangana, India

**Keywords:** Newcastle disease, lung, Leghorn and Fayoumi, long non-coding RNAs, differential resistance, coexpression network, qPCR validation

## Abstract

**Introduction:**

Newcastle disease is one of the significant issues in the poultry industry, having catastrophic effects worldwide. The lung is one of the essential organs which harbours Bronchus-associated lymphoid tissue and plays a vital role in the immune response. Leghorn and Fayoumi breeds are known to have differences in resistance to Newcastle disease. Along with genes and long non-coding RNAs (lncRNAs) are also known to regulate various biological pathways through gene regulation.

**Methods:**

This study analysed the lung transcriptome data and identified the role of genes and long non-coding RNAs in differential immune resistance. The computational pipeline, FHSpipe, as used in our previous studies on analysis of harderian gland and trachea transcriptome was used to identify genes and lncRNAs. This was followed by differential expression analysis, functional annotation of genes and lncRNAs, identification of transcription factors, microRNAs and finally validation using qRT-PCR.

**Results and discussion:**

A total of 8219 novel lncRNAs were identified. Of them, 1263 lncRNAs and 281 genes were differentially expressed. About 66 genes were annotated with either an immune-related GO term or pathway, and 12 were annotated with both. In challenge and breed-based analysis, most of these genes were upregulated in Fayoumi compared to Leghorn, and in timepoint-based analysis, Leghorn challenge chicken showed downregulation between time points. A similar trend was observed in the expression of lncRNAs. Co-expression analysis has revealed several lncRNAs co-expressing with immune genes with a positive correlation. Several genes annotated with non-immune pathways, including metabolism, signal transduction, transport of small molecules, extracellular matrix organization, developmental biology and cellular processes, were also impacted. With this, we can understand that Fayoumi chicken showed upregulated immune genes and positive cis-lncRNAs during both the non-challenged and NDV-challenge conditions, even without viral transcripts in the tissue. This finding shows that these immune-annotated genes and coexpressing cis-lncRNAs play a significant role in Fayoumi being comparatively resistant to NDV compared to Leghorn. Our study affirms and expands upon the outcomes of previous studies and highlights the crucial role of lncRNAs during the immune response to NDV.

**Conclusion:**

This analysis clearly shows the differences in the gene expression patterns and lncRNA co-expression with the genes between Leghorn and Fayoumi, indicating that the lncRNAs and co-expressing genes might potentially have a role in differentiating these breeds. We hypothesise that these genes and lncRNAs play a vital role in the higher resistance of Fayoumi to NDV than Leghorn. This study can pave the way for future studies to unravel the biological mechanism behind the regulation of immune-related genes.

## Introduction

Newcastle disease is one of the significant issues in the poultry industry, having catastrophic effects worldwide, especially in emerging nations. It is a highly contagious viral disease of Avians caused by the Newcastle disease virus (NDV), a ss-RNA virus belonging to the Paramyxoviridae family (Ganar et al., 2014). This is endemic in most of Asia, Africa and some parts of North and South America. The general approach to controlling the disease is vaccination, which would result in immunity against NDV infection and virus replication (Mebrate et al., 2019). Although vaccination is the most effective treatment available currently, the lack of suitable infrastructure in developing countries, the development of new variants of the virus, lack of antigenically matched vaccines, improper immunisation and the existence of antibodies against vaccine strain led to the decreased efficacy of the administered vaccine (Dimitrov et al., 2017).

Leghorn and Fayoumi are the two inbred lines of chicken (*Gallus gallus*). While the Leghorn and Fayoumi breeds are known for their disease resistance, the Fayoumi breed is more resistant to Newcastle disease than the Leghorn breed (Vanamamalai et al., 2021; Vanamamalai et al., 2023; Venkata Krishna et al., 2024). In 2015, Jinxiu Li et al. performed bisulphite sequencing of Leghorn and Fayoumi chicken and identified differential methylation patterns in the genomic regions annotated with immune GO terms (Li et al., 2015). Melissa et al. have analysed the transcriptome response of Leghorn and Fayoumi chickens and showed genes playing essential roles in the differential resistance of these breeds (Deist et al., 2017). A study by MS Tarabany on immune response against Newcastle disease virus in Leghorn and Fayoumi breeds showed that in comparison to purebred Leghorn chicken, purebred Fayoumi and Fayoumi-Leghorn hybrid chicken had significantly higher antibody titers and lesser mortality rates during NDV infection (Mahmoud S, 2019). Additionally, Schilling et al. have explored chicken embryos and reported breed-specific expression of immune-related genes (Schilling et al., 2019). Besides NDV, Leghorn and Fayoumi showed differential resistance patterns against the Avian Influenza virus (Wang et al., 2014), *Eimeria* spp (Pinard-van der Laan et al., 2009). and *Salmonella* (Cheeseman et al., 2007). The genetic variations resulted in differences in the immune responses and susceptibility against various diseases, including NDV (Hassan et al., 2004).

Long non-coding RNAs (lncRNAs) are a type of RNA molecule that are longer than 200 nucleotides and do not code for proteins. They play essential roles in various biological processes, including gene regulation, chromatin remodelling, and cell differentiation. They can act as transcriptional regulators by interacting with DNA, RNA, and proteins to modulate gene expression. They can also act as scaffolds for protein complexes, combining multiple proteins to perform specific functions. Recent studies have suggested that lncRNAs are involved in various diseases, including cancer, neurological disorders, and cardiovascular disease. Because of their diverse functions and potential roles in various diseases, there is a potential for understanding the role of lncRNAs during Newcastle disease in chickens. In our previous studies, the role of lncRNAs was studied during Newcastle disease in the harderian gland (Vanamamalai et al., 2021; Venkata Krishna et al., 2024) and trachea (Vanamamalai et al., 2023) transcriptome data. These studies have identified several cis-lncRNAs that co-express with immune-related genes. These were found to be upregulated in Fayoumi when compared to Leghorn. In addition, Leghorn chickens have shown downregulation of immune-related genes during the progress of ND (between time points), which was not identified in Fayoumi.

Lung tissue plays a vital role in the immune response, mainly in case of respiratory infection like NDV, as the lung hosts one of the critical immune tissues, Bronchus-associated lymphoid tissue (BALT), which produces a variety of immune cells, including B cells and T cells (Hwang et al., 2016). Although Leghorn and Fayoumi are breeds of the same species, there are differences in their resistance and immune response against NDV. This study hypothesises that some immune-related genes might play a vital role in these differential resistance patterns. Different lncRNAs might play a significant role in regulating these immune-related genes, thus making Fayoumi comparatively resistant to NDV compared to Leghorn. With this, the current study aims to investigate lung tissue transcriptome data to identify the potential lncRNAs, understand the differences in the expression of these lncRNAs and genes between challenged and non-challenged chicken, between the two breeds and during the progress of infection, identify the co-expression between genes and lncRNAs and determine the roles of the lncRNAs using the functional annotation of the genes co-expressing with the lncRNAs, which helps in understanding the role of long non-coding RNAs in the lung during Newcastle disease challenge.

## Methods

### Data collection

The transcriptome sequencing dataset was downloaded from a publicly available resource – EBI ENA, from the project PRJEB21760 (Deist et al., 2017). This dataset consists of 48 samples of chicken lung tissue sequenced using Illumina Hiseq 2500 with single-end 100bp reads. Of these 48 samples, 24 belong to the Leghorn breed, and 24 belong to the Fayoumi breed. Regarding infectivity, 12 out of 24 samples of each breed were control non-challenged samples, and the other 12 were NDV-challenged samples. This dataset includes three different time points concerning the viral challenge – 2 DPC (days post-challenge), 6 DPC and 10 DPC. The details of the downloaded data are mentioned in **Supplementary Table 1**.

### Identification of lncRNAs – FHSpipe

In this study, the pipeline described in our previous study (Vanamamalai et al., 2023) was used to identify lncRNAs, perform differential expression and further downstream analysis. This pipeline includes different steps like quality check which was performed using Fastp tool (Chen et al., 2018), followed by mapping of clean reads against the latest Chicken reference genome of version GRCg7b using the tool Hisat2 v2.2.1 (Kim et al., 2019) followed by assembly of mapped reads into transcripts using Stringtie v2.1.4 (Pertea et al., 2015), annotation of the transcripts into different class codes using GFFCompare (Pertea and Pertea, 2020), extraction of the sequences of transcripts annotated with class codes “I” (intronic), “U” (unknown/intergenic) and “X” (anti-sense) and filtering these sequences by eliminating the sequences with a length less than 200 nucleotides, ORF length greater than 100 amino acids (300 nucleotides), having hits against protein family (Pfam) database and sequences annotated with “coding” tag using Coding potential calculator 2 (CPC2) (Kang et al., 2017). The filtered sequences were finally subjected to BLAST against various databases like NONCODE v6 (Zhao et al., 2021) to identify known and novel lncRNAs, tRNA database (Chan and Lowe, 2009), SILVA rRNA database (Quast et al., 2013) and miRbase (Kozomara et al., 2019) to eliminate other non-coding RNAs, if any, and the finally remaining sequences were considered as potential long non-coding RNAs.

### Differential expression analysis

To perform differential expression analysis of genes and the identified lncRNAs, FHSpipe generates read counts for genes and lncRNAs, which can be used to perform differential expression analysis using edgeR v3.34.1 (Robinson et al., 2010) with the generalised linear model (GLM). Three factors were considered while writing the contrasts as used in our previous study (Vanamamalai et al., 2023) – challenge (challenged and non-challenged), breed (Leghorn and Fayoumi) and timepoint (2, 6, 10). Differential expression was performed in three cases, and heatmaps were plotted for both genes and lncRNAs using gplots R library (Warnes et al., 2020). The identified differentially expressed genes (DEGs) and lncRNAs (DElncRNAs) were plotted against chromosomes based on their position using Circos (Krzywinski et al., 2009).

### Gene ontology analysis and GSEA

As described earlier, OmicsBox v2.1.14 (BioBam Bioinformatics, 2019) was used to perform the GO functional annotation and the pipeline contains six steps – BLAST (Altschul et al., 1990) against nonredundant (nr) protein database, Interpro scan (Philip et al., 2014) against various protein databases, Gene ontology mapping (Gotz et al., 2008) against GOA version 2022.03, GO annotation (Gotz et al., 2008), mapping against EggNOG v5.0.2 database (Huerta-Cepas et al., 2019) and KEGG (Kanehisa and Goto, 2000) and Reactome (Fabregat et al., 2018) combined pathway analysis. The Gene Set Enrichment Analysis (GSEA) module in OmicsBox (Subramanian et al., 2005) was used to perform GO functional enrichment analysis to identify enriched gene ontologies (“Biological Process”, “Molecular Function”, and “Cellular Component”) and enriched pathways (“Reactome” and “KEGG”). The parameters, including weighted enrichment statistic, the gene set permutation number and other filters, were set as mentioned earlier (Vanamamalai et al., 2023).

### Functional annotation of lncRNAs

The functions of the Co-expressing genes were used to predict the functions of the lncRNAs by subjecting the co-expressing gene-lncRNA pairs to cis-trans analysis (Guttman and Rinn, 2012; Zhao et al., 2020). The pairs with lncRNA and gene on the same chromosome were termed cis, and the pairs with lncRNA and gene on different chromosomes were termed trans. WGCNA (Weighted Gene Correlation Network Analysis) v1.70-3 (Langfelder and Horvath, 2008) was used to analyse the co-expression of the differentially expressed genes and lncRNAs. As mentioned in the tutorials on the WGCNA webpage, the pipeline was followed to perform co-expression analysis. The expression values (FPKM) of the genes and lncRNAs were used as input. All the parameters, including outlier height cut-off and minimum module size, were selected based on the input data, and a scale-free topology fit index cut-off of 0.8 was used to select soft power. The eigengenes module was used to merge the closely related modules. The text files containing the data regarding the edges and nodes of the co-expression network were extracted as final output.

### Gene-transcription factor interaction analysis

The transcription factors interacting with the genes at 5’ UTRs were identified using the MEME toolkit version 5.4.1 (Bailey et al., 2009). This pipeline includes two steps – identification of motifs and identification of transcription factors of the obtained motifs. First, motifs were identified using the MEME tool and then obtained motifs were compared against the JASPAR 2023 vertebrate database using the TomTom tool. The identified transcription factors were searched against the Animal TF database v4.0 (Shen et al., 2023) to obtain Chicken Transcription factors.

### Gene -miRNA interaction analysis

The online server miRNet (Chang et al., 2020) was used to obtain the microRNAs targeting the differentially expressed genes obtained in all three conditions. The official gene symbol of these genes was used to search the microRNAs targeting them. A list of microRNAs was downloaded in the form of a comma-separated file.

### Network visualisation

The data, including co-expressing pairs of genes and long non-coding RNAs, transcription factors interacting with 5’ UTR of the DEGs and microRNAs targeting these DEGS, was used to construct a network and plotted using Cytoscape v3.10 (Shannon et al., 2003). The differentially expressed genes annotated with immune-related level 2 Gene ontologies, i.e., response to biotic stimulus (GO:0009607) and immune system process (GO:0002376), and Reactome Immune system pathways were selected for visualisation. Accordingly, the data of long non-coding RNAs, Transcription factors and microRNAs was filtered.

### QTL analysis

The quantitative trait loci information of the differentially expressed genes was obtained from the chicken QTL data available on the Animal QTL database (Hu et al., 2019). The data was downloaded in GFF format, and the QTL information was acquired using an in-house Python script.

### Validation studies

Validation of 4 selected DElncRNAs with co-expressing genes for 3 lncRNAs was performed as described earlier (Vanamamalai et al., 2023), including three pairs of co-expressing lncRNA-genes, namely TCONS_00098393 (Lnc 1) – IFIT5 (Gene 1), TCONS_00133885 (Lnc 2) – CD55 (Gene 2), TCONS_00381885 (Lnc 3) – LOC112533599 (Gene 3) and one lncRNA without co-expressing gene – TCONS_00383493 (Lnc 4). All the experiments were performed according to the guidelines approved by the Institute Animal Ethics Committee (IAEC/DPR/20/2). The detailed information on the Primers generated using NCBI Primer-BLAST (Ye et al., 2012) is mentioned in **Supplementary Table 2**. Briefly, chickens (aged 21 days) were chosen for the study. LaSota strain of NDV of 200μL of EID 50 ≥ 10^6^ per dose was used for inoculation, and phosphate-buffered saline was inoculated for the control group. The viral antibody levels were determined as described earlier (Vanamamalai et al., 2023) using Hemagglutination inhibition (HI) and indirect ELISA. Lung tissue was collected at three time points – 2, 6 and 10. A viral RNA purification kit (Himedia Pvt. LTD) was used for total RNA isolation, High-capacity cDNA Reverse transcription Kit (Applied Biosystems, USA) was used for cDNA reverse transcription and real-time PCR was performed using Maxima SYBR Green/ROX qPCR Master Mix (2X) (MBI Fermentas, USA) on Insta Q96™ real-time PCR machine (Himedia, India) machine. The 2^-ΔΔCt method was utilised to calculate relative expression between respective conditions (1 DEG and 2 DElncRNAs at 2 DPC and 2 DEGs and 2 DElncRNAs at 6 DPC). The results were visualised as bar plots with error bars (SEM) and significance values from multiple unpaired t-tests, plotted using GraphPad Prism^1^.

## Results

### Data preprocessing

The quality assessment of all the samples using the Fastp tool showed that an average of 97.2% of the reads (94.5-97.8%) of all 48 samples passed the quality filter with a quality cut-off of 25. About 2.65% of reads were discarded due to low quality, 0.01% were discarded due to too many ‘N’ bases, and 0.14% were too short. The average GC content was 48.5%, and the average Q30 base content post-filtering was about 95.02%. The detailed result for each of the 48 samples was shown in **Supplementary Table 3**.

### Mapping and assembly

The cleaned high-quality reads were mapped against the latest version of the chicken reference genome, i.e., GRCg7b. The mapped reads were assembled into potential transcripts with the help of reference annotation. As mentioned in **Supplementary Table 3**, the average mapping percentage across all 48 samples was 94.6%, with the sample mapping in the range of 92.78% to 95.84%. Post assembly, the average number of transcripts was found to be 43904, ranging from 42729 to 46501. After reassembly, about 25451 transcripts were obtained in each sample.

### Identification of long non-coding RNAs

**Figure 1:**
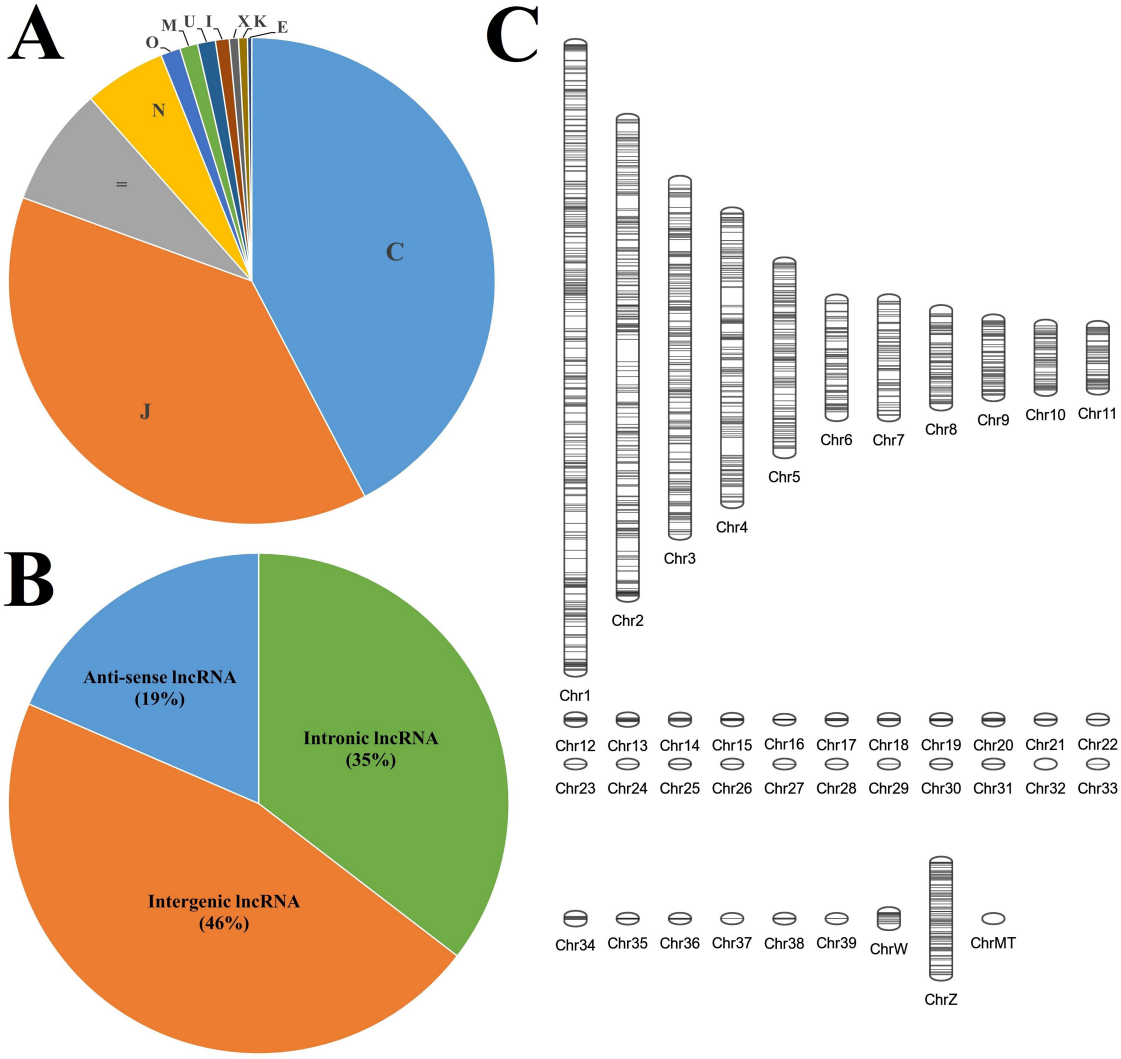
The characteristics of the long non-coding RNAs identified in the lung transcriptome. **(A)** Class code classification of all the transcripts. **(B)** Pie chart composition of different types of lncRNAs. **(C)** Chromosomal localisation of the identified lncRNAs.

A total of 415627 transcripts were annotated with 11 different class codes, with the class code C (transcripts contained in reference) being annotated to the highest number of transcripts (42.3%). A total of 11216 transcripts were found to be annotated with Class codes U with the highest transcripts (4914, 44%) followed by I (3780, 34%) and X with the least transcripts (2522, 22%), as shown in **Figure 1A**. The 11216 transcripts representing class codes I, U, and X were subjected to various filtration steps, and 2997 transcripts were eliminated, with 8219 transcripts being extracted as final potential long non-coding RNAs. These details are mentioned in **Table 1**. Of these, the highest number of transcripts, i.e., 3788 (46%), were found to be intergenic (U), followed by intronic (I), i.e., 2911 (35%), and anti-sense (X) with the least transcripts, i.e., 1520 (19%), as shown in **Figure 1B**. Of the 8219 transcripts, 5532 showed no hits against the NONCODE v6 database, indicating novel transcripts and 2687 showed similar hits. Of these, 471 showed 100% similarity, and only 6 showed 100% similarity and 100% coverage. There were no hits with 100% similarity and 100% coverage against miRBase (Mature and Hairpin) and transfer RNA database. The chromosomal positions of these lncRNAs were plotted using the Phenogram tool and the plot was shown in **Figure 1C**. All the chromosomes showed lncRNAs except Chromosome 32, which had no lncRNAs mapped. Chromosome 1 showed the highest percentage of lncRNAs, and Chromosome 33 showed the lowest percentage.

**Table 1:** The statistics of the lncRNA identification pipeline.

STEP	Number of sequences eliminated	Number of sequences retained
Total	–	11216
Length filter (<200nt)	0	11216
ORF filter (>100aa)	1767	9449
Pfam filter	179	9270
CPC2	1051	8219
Final	2997	8219

### Differential expression analysis

**Figure 2:**
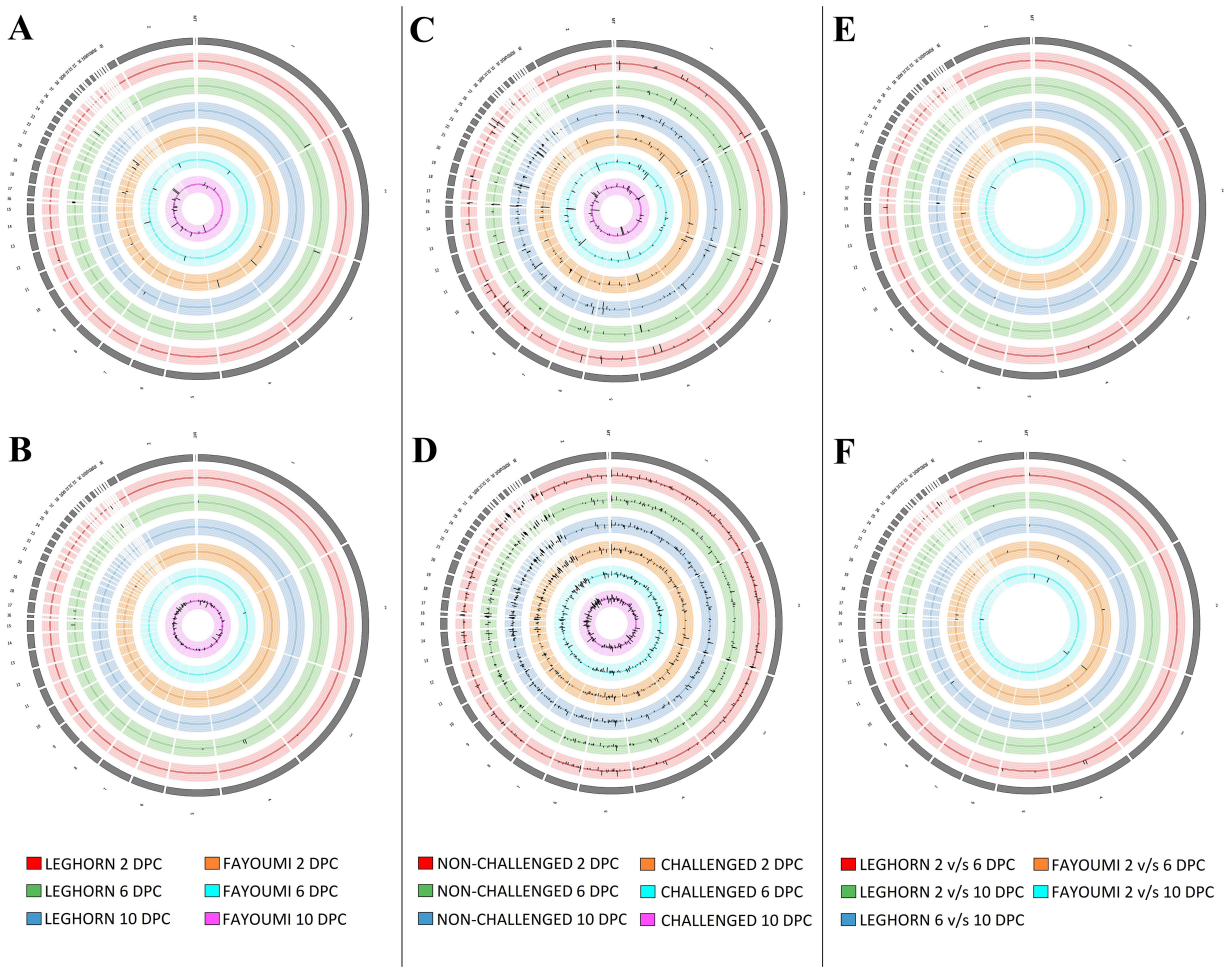
The synteny plot of the Chromosomal localisation of differentially expressed genes **(A, C, E)** and differentially expressed lncRNAs **(B, D, F)** identified in challenge-based analysis **(A, B)**, breed-based analysis **(C, D)** and timepoint-based analysis **(E, F)**.

Of the three-factor analyses used for estimation of differential expression, about 83 DEGs and 183 DElncRNAs were identified in the challenge-based analysis, i.e., comparison between challenged and non-challenged chicken, about 261 DEGs and 1165 DElncRNAs in breed-based analysis, i.e., comparison between Leghorn and Fayoumi, and about 47 DEGs and 55 DElncRNAs in timepoint-based analysis, i.e., comparison between different timepoints. The number of differentially expressed genes and lncRNAs identified in each of the three analysis conditions were mentioned in **Table 2** – A: challenge-based analysis, B: breed-based analysis and C: timepoint-based analysis. Heatmaps showing the read count data were shown in **Supplementary Figure 1** for genes and **Supplementary Figure 2** for lncRNAs. The characteristics and expression values of the extracted lncRNAs are mentioned in **Supplementary Table 4**. **Figure 2** represents the chromosomal localisation of the DEGs and DElncRNAs identified in the three analysis factors - challenge (A, B), breed (C, D) and timepoint (E, F). The outermost grey circle represented the chromosomes of chickens, followed by the circle showing the genes (A, C, E) and lncRNAs (B, D, F) of the different conditions as mentioned in the legend. This figure shows that more differentially expressed genes and lncRNAs were obtained in breed-based analysis compared to the challenge-based and timepoint-based analysis. In comparison to genes, a more significant number of lncRNAs were observed. In challenge-based analysis, Leghorn 6 DPC showed a higher number of genes and Fayoumi 10 DPC showed a higher number of lncRNAs. In breed-based analysis, non-challenged 10 DPC showed a higher number of genes and lncRNAs. In timepoint-based analysis, Leghorn 6 v/s 10 DPC showed a higher number of genes and Leghorn 2 v/s 6 DPC showed a higher number of lncRNAs.

**Table 2:** The number of differentially expressed genes and long non-coding RNAs identified in (A) – Challenge-based analysis, (B) – Breed-based analysis and (C) – Timepoint-based analysis.

A	Leghorn			Fayoumi		
	2-day	6-day	10-day	2-day	6-day	10-day
**DEGs**	2	33	1	12	5	31
**DElncRNAs**	2	10	0	1	2	168
B	Non-challenged			Challenged		
	2-day	6-day	10-day	2-day	6-day	10-day
**DEGs**	75	60	162	60	79	45
**DElncRNAs**	530	580	727	568	422	394
C	Leghorn			Fayoumi		
	2v6	2v10	6v10	2v6	2v10	6v10
**DEGs**	6	2	34	0	5	3
**DElncRNAs**	34	7	4	0	13	6

### Gene ontology analysis

**Figure 3:**
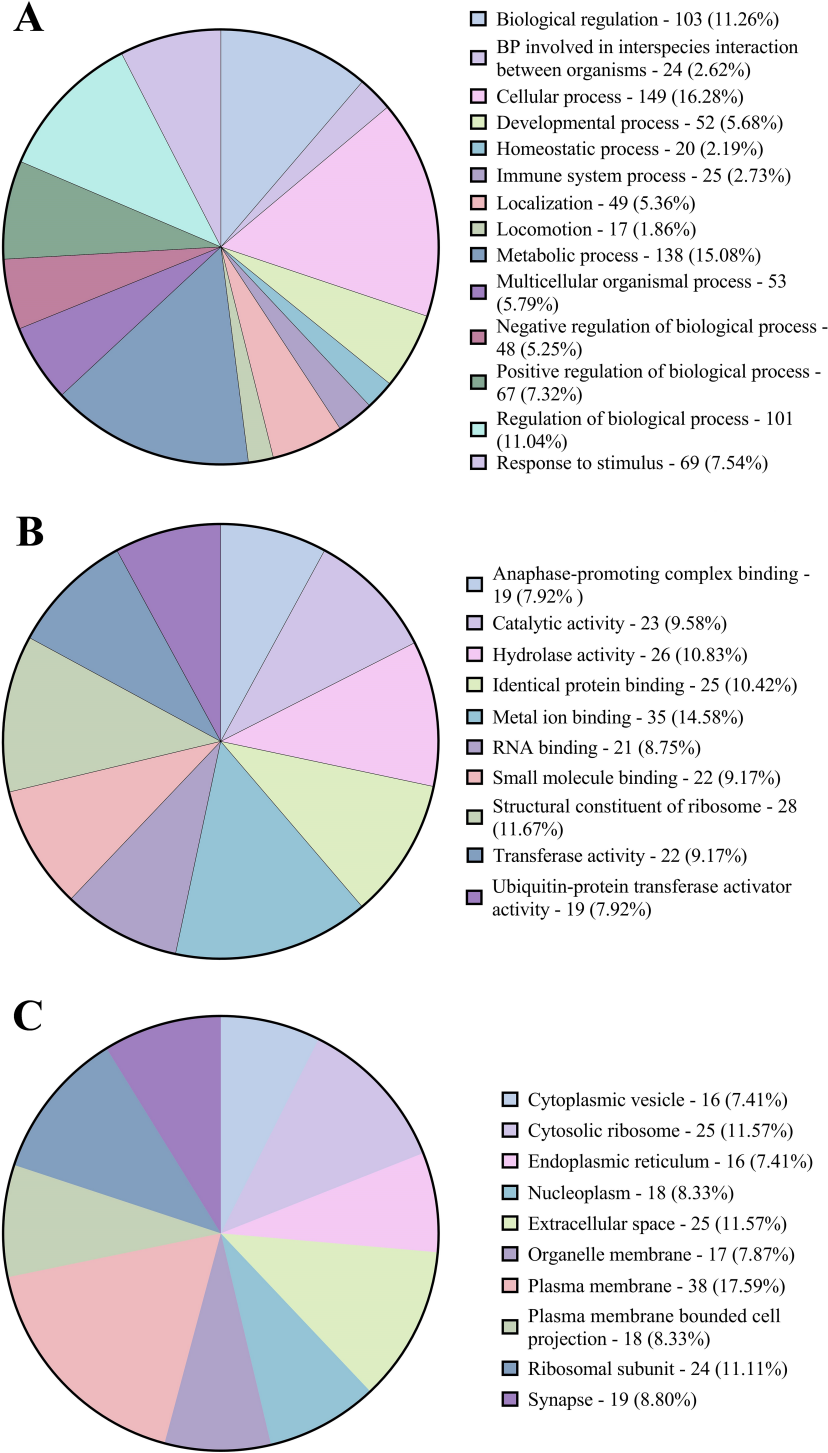
Pie charts representing **(A)** the proportions of Level 2 biological process gene ontologies. **(B)** the distribution of molecular function gene ontologies. **(C)** the distribution of cellular component gene ontologies.

**Figure 4:**
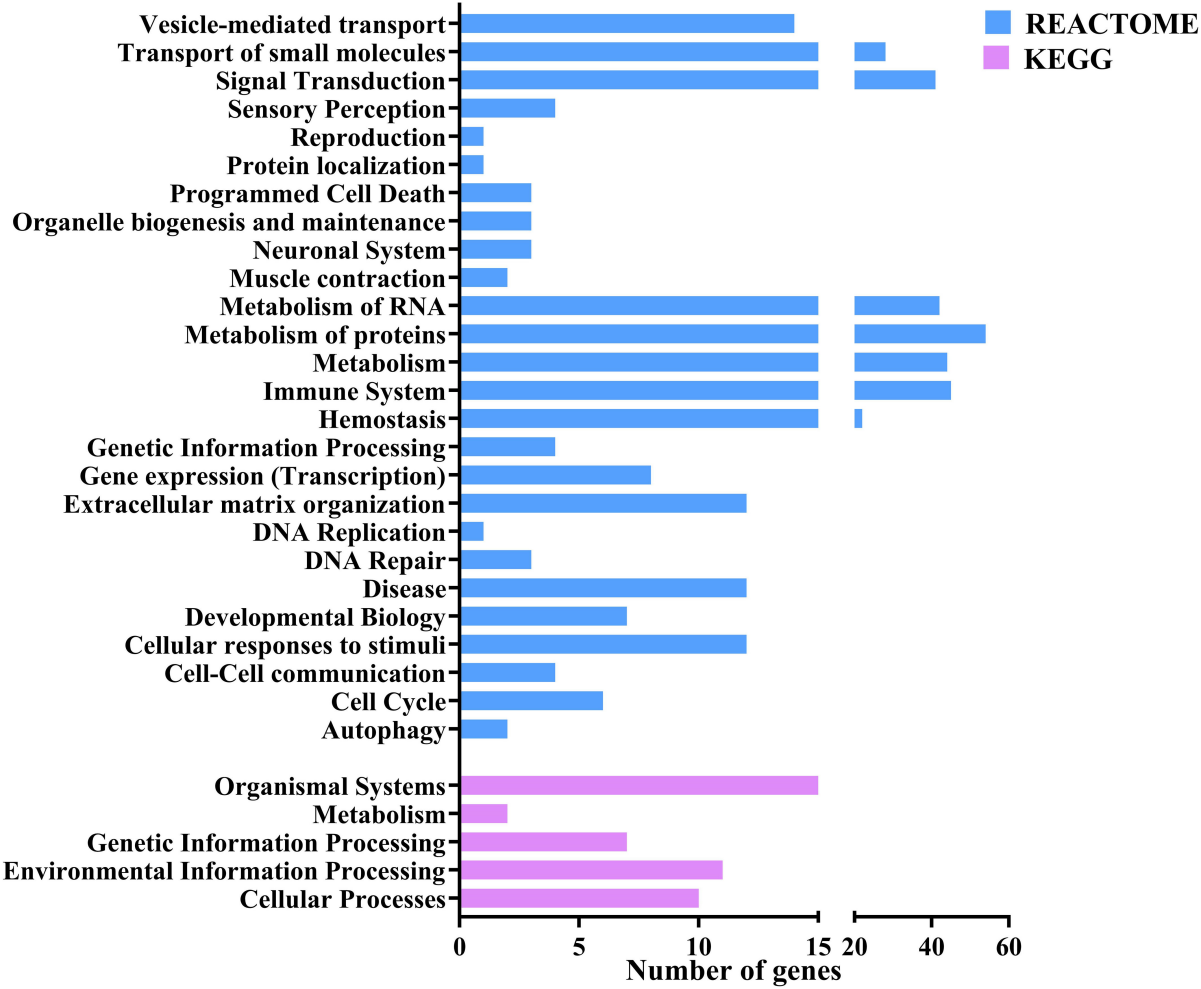
Bar plot representing the number of the differentially expressed genes annotated to different pathways across various categories of KEGG and Reactome databases.

Gene ontology (GO) and functional annotation results were obtained in various forms including biological process, molecular function, cellular component GOs, and KEGG and Reactome pathways. The GO annotation results were plotted as pie charts using GraphPad Prism (GraphPad Software) and were represented in **Figure 3** with (A) Level 2 biological process GOs, (B) sequence distribution of molecular function GOs and (C) sequence distribution of cellular component GOs. A total of 14 different biological process level 2 GOs were obtained, of which cellular process (GO:0009987) was annotated to the highest number of genes, i.e., 149 (16.28%). In contrast, locomotion (GO:0040011) was annotated to the lowest number of genes, i.e., 17 (1.78%). Also, about 25 (2.61%) genes were annotated with the immune system process (GO:0002376). In addition, a total of 4 level 2 molecular function GOs were obtained, of which binding (GO:0005488) was annotated to the highest number of genes, i.e., 136 (51.32%) followed by catalytic activity (GO:0003824 – 25.94%), structural molecule activity (GO:0005198 – 11.65%) and molecular function regulator activity (GO:0098772) was annotated to lowest number of genes, i.e., 29 (10.94%). In the sequence distribution across ten molecular function GOs, the GO Metal ion binding (GO:0046872) was annotated to the highest percent of genes, i.e., 14.58%, as represented in **Figure 3B**. In the case of the cellular component, only 2 level 2 GOs were obtained, i.e., cellular anatomical entity (GO:0110165) was annotated to 158 (71.17%) genes and Protein-containing complex (GO:0032991) was annotated to 64 (28.83%) genes. In the sequence distribution of 10 cellular component GOs, the GO Plasma membrane (GO:0005886) was annotated to the highest percent of genes, i.e., 17.59% as represented in **Figure 3C**. **Figure 4** shows the distribution of the DEGs across different categories of KEGG and Reactome databases plotted using GraphPad Prism (GraphPad software). The KEGG database pathway analysis showed that most genes were annotated to pathways in the organismal systems category and very few pathways were annotated to the metabolism category. As per the Reactome database, most of the genes were annotated with pathways in the metabolism of proteins and only 1 pathway was found in the Protein localisation, Reproduction and DNA replication categories. A total of 45 different genes were annotated with pathways in the immune system (Reactome) category, which were annotated with different biological process GOs, including biological regulation (GO:0065007), cellular process (GO:0009987), immune system process (GO:0002376), response to biotic stimulus (GO:0009607) and Signalling (GO:0023052). Apart from this, 25 genes were annotated with the biological process GO - immune system process (GO:0002376), and 18 genes were annotated with the biological process response to biotic stimulus (GO:0009607). About 66 genes were annotated with either immune biological process GOs or pathways. Of this, 12 genes were annotated with both immune GOs and pathways. The detailed information on the functional annotation, including gene ontologies, pathways and the fold change values of all the DEGs, is mentioned in **Supplementary Table 5**.

### Functional enrichment analysis (GSEA)

Unlike the trachea (Vanamamalai et al., 2023), the lung showed enriched GOs and pathways under a few conditions. The number of total and enriched GOs and pathways are mentioned in **Table 3**. In challenge-based analysis, Only Leghorn 6 DPC showed enriched GOs. Under biological process, the highest number of enriched GOs were identified under level 2 GO developmental process (111), followed by biological regulation (61), cellular process (21), metabolic process (19), response to stimulus (15), signalling (6), localisation (2) reproductive process (2) and one each under multicellular organismal process and growth. In breed-based analysis, non-challenged 2 DPC showed no enriched GOs. Challenged 2 DPC showed the highest enriched GOs with biological process GOs under biological regulation (27), developmental process (11), cellular process (8), response to stimulus (8), metabolic process (4), localisation (3) and signalling (1). Challenge 6 DPC showed enriched GOs under biological regulation (7). Challenge 10 DPC showed enriched GOs under biological regulation (14) and developmental process (1). Non-challenged 6 DPC showed enriched GOs under metabolic process (2). Non-challenged 10 DPC showed enriched GOs under biological regulation (14), metabolic process (6), developmental process (4), localisation (3) and cellular process (1). In timepoint-based analysis, enriched GOs were obtained only in Fayoumi 2 vs 10 DPC, but none were identified under biological process. No enriched pathways were identified in the challenge-based analysis. In breed-based analysis, 11 pathways under disease (Reactome) were identified to be enriched in all six conditions. In addition to these 11 pathways, challenge 6 DPC showed enriched pathways under metabolism (KEGG) (1), non-challenge 2 DPC under Organelle biogenesis and maintenance (Reactome) (1), non-challenge 6 DPC under metabolism (KEGG) (2) and non-challenge 10 DPC under immune System (Reactome) (1) and metabolism of proteins (Reactome) (1). In timepoint-based analysis, only Fayoumi 2 vs 10 DPC showed enriched pathways under transport of small molecules (Reactome) (2). Detailed information on the enriched GOs and pathways is mentioned in **Supplementary Tables 6**, **7**, respectively.

**Table 3:** The number of Total and Enriched Gene Ontologies, i.e., Biological Process (BP), Molecular Function (MF) and Cellular Component (CC), and Pathways, i.e., KEGG and Reactome identified in all the conditions in (A) Challenge-based, (B) Breed-based and (C) Timepoint-based analysis.

A		LEGHORN			FAYOUMI		
		2-day	6-day	10-day	2-day	6-day	10-day
**BP**	**Total**	16	256	16	215	241	474
	**Enriched**	0	241	0	8	6	5
**MF**	**Total**	13	30	1	45	40	70
	**Enriched**	0	25	0	2	2	0
**CC**	**Total**	9	15	5	55	43	66
	**Enriched**	1	8	0	2	2	0
**KEGG**	**Total**	0	0	0	3	1	8
	**Enriched**	0	0	0	0	0	0
**REACTOME**	**Total**	6	15	0	16	20	84
	**Enriched**	0	0	0	0	0	0
B		NON-CHALLENGED			CHALLENGED		
		2-day	6-day	10-day	2-day	6-day	10-day
**BP**	**Total**	889	696	1202	693	687	551
	**Enriched**	0	2	28	64	7	17
**MF**	**Total**	157	107	212	114	124	105
	**Enriched**	0	0	6	5	0	0
**CC**	**Total**	132	126	185	132	142	124
	**Enriched**	0	1	0	4	0	1
**KEGG**	**Total**	35	18	70	27	12	24
	**Enriched**	0	2	0	0	1	0
**REACTOME**	**Total**	132	112	397	117	88	78
	**Enriched**	12	11	13	11	11	11
C		LEGHORN			FAYOUMI		
		2 v/s 6	2 v/s 10	6 v/s 10	2 v/s 6	2 v/s 10	6 v/s 10
**BP**	**Total**	263	16	44	0	131	191
	**Enriched**	0	0	0	0	0	0
**MF**	**Total**	31	11	10	0	33	24
	**Enriched**	0	0	0	0	1	0
**CC**	**Total**	16	4	2	0	27	26
	**Enriched**	0	0	0	0	0	0
**KEGG**	**Total**	0	0	0	0	2	0
	**Enriched**	0	0	0	0	0	0
**REACTOME**	**Total**	16	6	4	0	8	4
	**Enriched**	0	0	0	0	2	0

### Functional annotation of lncRNA

The co-expression analysis of differentially expressed genes and lncRNAs identified in three different conditions resulted in several modules. The parameters, including outliers, soft power, minimum size and number of modules before and after merging, and sizes of the top and least sized modules, were mentioned in **Supplementary Table 8**. The scale-free topology model fit cut-off was selected as 0.8, and the soft power of the conditions failing to reach the cut-off index for reasonable powers was chosen as 9. There were no outliers in the challenge-based analysis, and soft power was chosen as 9 for all conditions. There were no interactions at Leghorn 10DPC. In breed-based analysis, outliers were observed at 2DPC non-challenged and 6 DPC challenged conditions, and soft power was chosen as 15 and 12 for challenged 2 DPC and 10 DPC, respectively, as they have scale-free topology fit index values above 0.8. In contrast, in the case of other conditions, soft power was chosen as 9. In the case of timepoint-based analysis, outliers were found in 4 conditions and soft power was chosen as 9 for all the conditions. There were no interactions at Fayoumi 2v6 DPC. In the cis-trans analysis, 94.29% of the pairs were trans-acting, while 5.71% were cis-acting. Of these cis-acting pairs, 38.45% of pairs have lncRNA upstream of the gene, 59.36% have lncRNA downstream of the gene, 2.1% have lncRNA within a gene, 0.06% have lncRNA covering the 3’ end of the gene, and 0.04% have lncRNA covering 5’ end of the gene. The number of cis-trans pairs is mentioned in **Table 4**. The cis gene-lncRNA pairs were analysed further to predict the functions of these lncRNAs using the genes. A total of 745 DElncRNAs were found to be in 4490 cis-interactions with 168 DEGs. Of these interactions, a higher number of positive interactions, i.e., both genes and lncRNAs either upregulated or downregulated, were identified than negative interactions, i.e., genes and lncRNAs with one being upregulated and the other being downregulated. Of the 66 DEGs annotated with either immune GO or pathway, 46 DEGs were found to be co-expressing with 489 cis-DElncRNAs in a total of 975 interactions – 514 positive and 461 negative interactions. These details are mentioned in **Supplementary Table 9**.

**Table 4:** The number of Cis-Trans gene-lncRNA pairs and the different classes of Cis interacting pairs identified during - (A) Challenge-based analysis, (B) Breed-based analysis and (C) Timepoint-based analysis.

A		Leghorn			Fayoumi		
Timepoint		2 DPC	6 DPC	10 DPC	2 DPC	6 DPC	10 DPC
**Total interactions**		2	139	0	1	3	2640
**Trans**		2	138	0	1	3	2525
**Cis**	**Total**	0	1	0	0	0	115
	**Downstream**	0	1	0	0	0	62
	**Upstream**	0	0	0	0	0	53
	**Within gene**	0	0	0	0	0	0
	**At 3’ end**	0	0	0	0	0	0
	**At 5’ end**	0	0	0	0	0	0
B		Non-challenged			Challenged		
Timepoint		2 DPC	6 DPC	10 DPC	2 DPC	6 DPC	10 DPC
**Total interactions**		24328	22367	36676	10426	13450	10898
**Trans**		24326	20602	34399	9551	12435	10038
**Cis**	**Total**	2	1765	2277	875	1015	860
	**Downstream**	1	1076	1251	550	616	548
	**Upstream**	1	652	989	303	364	292
	**Within gene**	0	35	35	21	34	20
	**At 3’ end**	0	1	0	1	1	0
	**At 5’ end**	0	1	2	0	0	0
C		Leghorn			Fayoumi		
Timepoint		2v6	2v10	6v10	2v6	2v10	6v10
**Total interactions**		69	0	4	0	26	3
**Trans**		64	0	3	0	26	3
**Cis**	**Total**	5	0	1	0	0	0
	**Downstream**	0	0	0	0	0	0
	**Upstream**	4	0	1	0	0	0
	**Within gene**	0	0	0	0	0	0
	**At 3’ end**	1	0	0	0	0	0
	**At 5’ end**	0	0	0	0	0	0

### Gene-transcription factor interaction analysis

A total of 10 different motifs were identified as per the cut-off mentioned in the methods section. Of this, 8 motifs showed hits with transcription factors. With a q-value cut-off (<0.05), a total of 3 motifs (1, 4, 6) and 34 transcription factors were obtained. Motif 1 was found to be interacting with 1 transcription factor, Motif 4 with 12 transcription factors and Motif 6 with 32 transcription factors. These transcription factors belong to three families - E2F, zf-C2H2 and ZBTB. Most of them belong to the zf-C2H2 family. The list of motifs, their transcription factors, family and other details are mentioned in **Supplementary Table 10A** and details of transcription factors associated with immune-related genes were mentioned in **Supplementary Table 10B**.

### Gene -miRNA interaction analysis

A total of 504 different microRNAs were identified, which target 169 different genes. About 14 miRNAs were found to target immune related genes. Similar to our previous report on the trachea transcriptome (Vanamamalai et al., 2023), the gene BAIAP2 was found to be targeted by 78 different miRNAs and the miRNA gga-mir-1587 was found to target 23 different DEGs. The details of microRNAs associated with DEGs were mentioned in **Supplementary Table 10C**.

### Network visualisation

**Figure 5:**
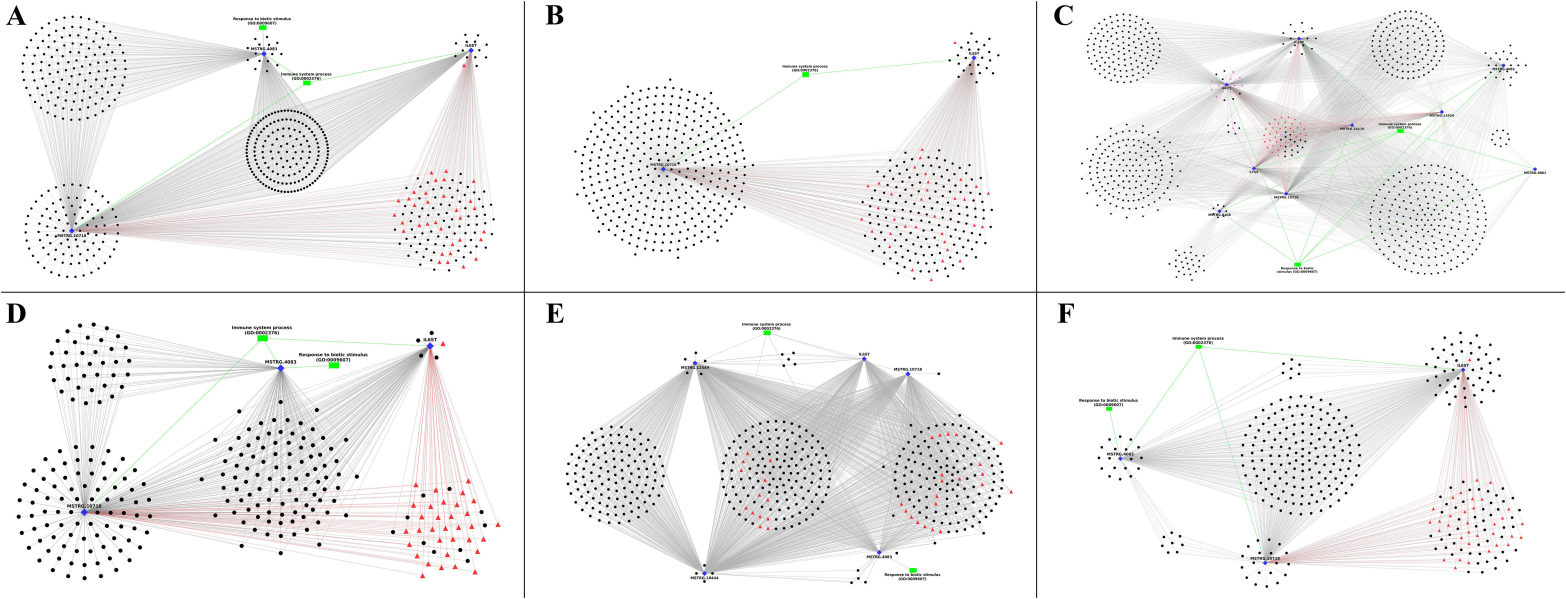
The plot of the network between differentially expressed genes (blue colour diamond shaped), co-expressing differentially expressed lncRNAs (black colour spherical shaped), transcription factors (red colour triangular shaped), microRNAs (pink colour arrow shaped) and biological process GOs (green colour rectangular shaped) identified in breed based analysis. **(A)** – non-challenged 2 days, **(B)** – non-challenged 6 days, **(C)** – non-challenged 10 days, **(D)** – challenged 2 days, **(E)** – challenged 6 days, **(F)** – challenged 10 days.

The networks of the selected immune-related DEGs, co-expressing DElncRNAs, transcription factors, microRNAs, and biological process level 2 GOs were constructed and visualised in the form of networks and plotted using Cytoscape. There were no interactions involving immune-related genes in challenge-based and timepoint-based analysis. **Figure 5** shows the network plots for breed-based analysis. The black circular nodes denote long non-coding RNAs, blue diamond nodes denote genes, green boxes denote Gene ontology (Biological process Level 2), pink arrows denote microRNAs targeting the genes, and red triangles denote Transcription factors binding to genes. **Figure 5A** shows the network obtained with non-challenged 2 DPC data with three DEGs annotated with two BP GOs, 511 lncRNAs and 34 TFs. No miRNAs were identified. Of the three DEGs, the gene MSTRG.10718 showed interactions with the highest number of lncRNAs (483), while the gene IL6ST showed the least (268). A total of 124 lncRNAs were identified to be explicitly interacting with a single gene, i.e., MSTRG.10718 (96), MSTRG.4083 (15) and IL6ST (13). **Figure 5B** shows the network obtained with non-challenged 6 DPC data with two DEGs annotated with one BP GO, 541 lncRNAs and 34 TFs. No miRNAs were identified. Of the two DEGs, the gene MSTRG.10718 showed the highest interactions (516), while the gene IL6ST showed the least (188). A total of 378 lncRNAs were identified to be explicitly interacting with a single gene, i.e., MSTRG.10718 (353) and IL6ST (25). **Figure 5C** shows the network obtained with non-challenged 10 DPC data with nine DEGs annotated with two BP GOs, 718 lncRNAs and 34 TFs. No miRNAs were identified. Of the two DEGs, the gene IL6ST showed the highest interactions (557), while the gene MSTRG.9061 showed the least (15). A total of 52 lncRNAs were identified to be explicitly interacting with a single gene, i.e., MSTRG.4083 (23), IL6ST (15), MSTRG.8108 (7), NPC2 (6) and MSTRG.14170 (1). **Figure 5D** shows the network obtained with challenged 2 DPC data with three DEGs annotated with two BP GOs, 235 lncRNAs and 34 TFs. No miRNAs were identified. Of the three DEGs, the gene MSTRG.10718 showed the highest interactions (231), while the gene IL6ST showed the least (112). A total of 90 lncRNAs were identified to be explicitly interacting with a single gene, i.e., MSTRG.10718 (86) and IL6ST (4). **Figure 5E** shows the network obtained with challenged 6 DPC data with five DEGs annotated with two BP GOs, 412 lncRNAs and 34 TFs. No miRNAs were identified. Of the five DEGs, the genes MSTRG.12549 and MSTRG.18444 showed the highest interactions (400), while the gene MSTRG.4083 showed the least (108). A total of 11 lncRNAs were identified to be explicitly interacting with a single gene, i.e., MSTRG.12549 (5), MSTRG.18444 (4), MSTRG.10718 (1) and MSTRG.4083 (1). **Figure 5F** shows the network obtained with challenged 10 DPC data with three DEGs annotated with two BP GOs, 311 lncRNAs and 34 TFs. No miRNAs were identified. Of the three DEGs, the gene IL6ST showed the highest interactions (266), while the gene MSTRG.4083 showed the least (193). A total of 86 lncRNAs were identified to be explicitly interacting with a single gene, i.e., IL6ST (49), MSTRG.10718 (19) and MSTRG.4083 (18).

### QTL analysis

The quantitative trait loci of the DEGs were identified using the gene coordinates, and different types of QTLs, including exterior, physiological, production, and health, were identified. The number of genes under each category of QTLs is shown in **Table 5**. Challenge-based analysis showed a higher number of QTLs, and the Production QTL category had the highest number of genes.

**Table 5:** The number of differentially expressed genes associated with different types of QTL obtained in (A) Challenge-based analysis, (B) Breed-based analysis and (C) Timepoint-based analysis.

A	Leghorn			Fayoumi		
	2 DPC	6 DPC	10 DPC	2 DPC	6 DPC	10 DPC
Total Genes	2	7	1	6	3	25
Exterior QTL	0	1	1	2	1	11
Health QTL	1	5	0	3	2	12
Physiology QTL	1	0	0	1	3	11
Production QTL	1	3	1	6	3	25
B	Non-challenged			Challenged		
	2 DPC	6 DPC	10 DPC	2 DPC	6 DPC	10 DPC
Total Genes	54	45	123	44	36	32
Exterior QTL	26	21	45	20	14	11
Health QTL	31	26	58	26	22	17
Physiology QTL	18	16	45	16	12	12
Production QTL	53	42	118	43	30	29
C	F2v6	F2v10	F6v10	L2v6	L2v10	L6v10
Total Genes	0	3	1	4	2	7
Exterior QTL	0	0	0	3	0	1
Health QTL	0	3	1	3	1	5
Physiology QTL	0	0	1	1	1	0
Production QTL	0	3	1	4	1	2

### Validation studies

**Figure 6:**
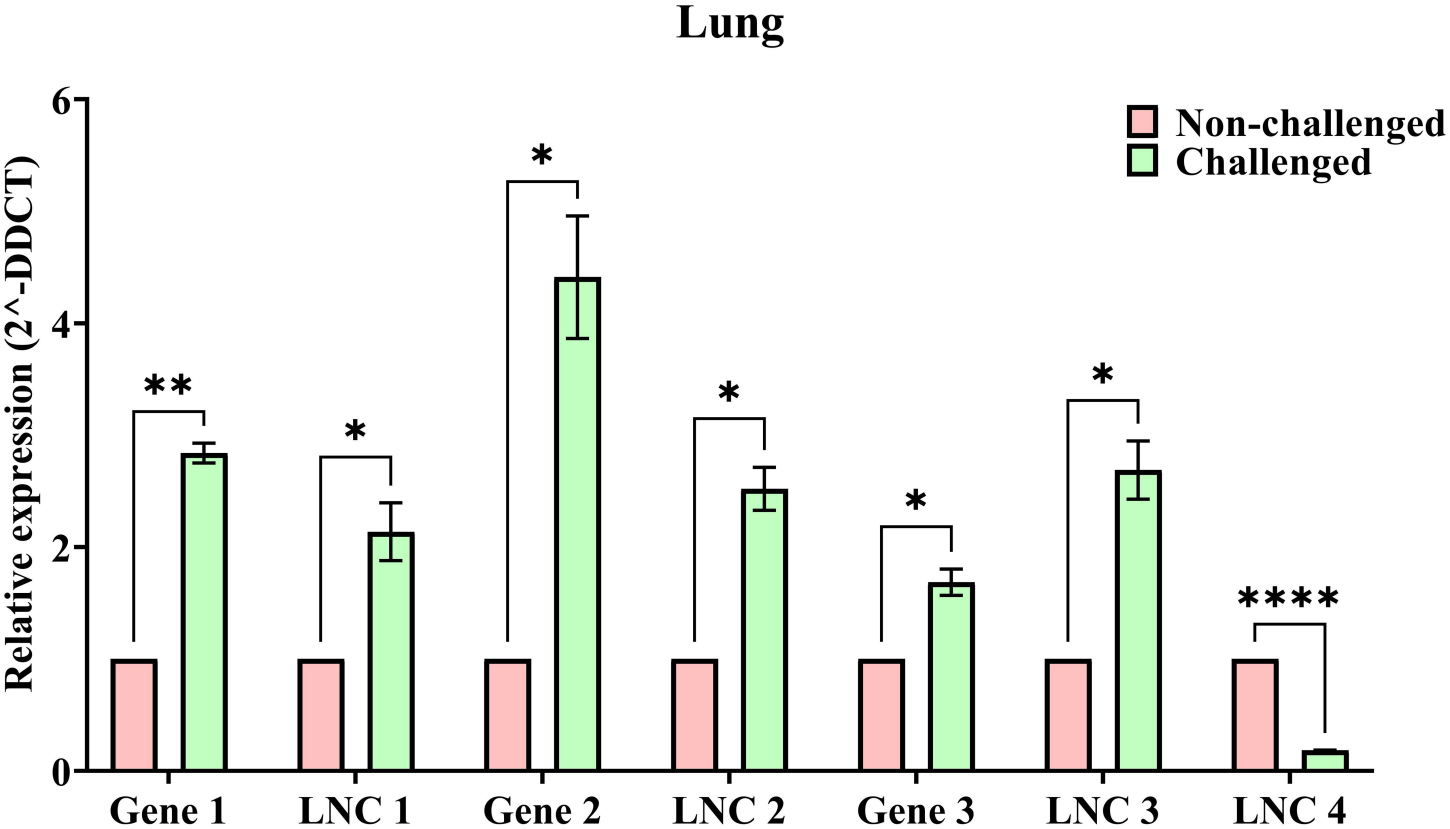
The bar plots representing the relative expression (2^-DDCT) of NDV challenged (Green bars) and Non-challenged (Red bars) samples with error bars representing the SEM values and significance (p) values: * - p <= 0.05, ** - p <= 0.01, **** - p <= 0.0001.

**Figure 6** shows the relative expression values between challenged (green bars) and non-challenged (red bars) of the selected 4 DElncRNAs and 3 co-expressing DEGs. The relative expression values were similar to those obtained from in silico analysis. The expression levels of the lncRNAs and the co-expressing genes were also validated, indicating the validation of the co-expression of these lncRNAs and genes. The significance values calculated using the unpaired t-test represent –*: p <= 0.05, **: p <= 0.01, ****: p <= 0.0001. All the genes and lncRNAs were found to be significant.

## Discussion

Newcastle disease is one of the challenging diseases of chickens, devastatingly impacting poultry industries, especially in underdeveloped countries. Along with the trachea, the lung is one of the essential tissues during respiratory infections due to the presence of Bronchus-associated lymphoid tissue (BALT). Previously, analysis of the harderian gland and trachea transcriptome has shown differences in the expression patterns of genes and co-expressing long non-coding RNAs, which were involved in a wide range of biological processes and pathways, including Immune-system related pathways. These studies have shown that although Leghorn showed upregulated immune genes in challenge-based analysis, Fayoumi was found to be showing higher upregulation in immune genes than Leghorn in breed-based analysis. In addition, Leghorn showed downregulation of immune genes in timepoint-based analysis, while Fayoumi showed no such response.

In the current study, investigating the lung tissue transcriptome has given essential insights into the immune response of Leghorn and Fayoumi chicken during Newcastle Disease. Several novel lncRNAs were identified, co-expressing with different genes involved in immune system pathways. A higher number of intergenic lncRNAs, also known as long intergenic lncRNAs (lincRNAs), were identified in comparison to intronic and anti-sense lncRNAs. Breed-based analysis showed more DEGs and DElncRNAs than challenge-based and timepoint-based analysis. Non-challenged datasets showed more DEGs and DElncRNAs than challenged datasets, which shows the differences in the expression of genes between Leghorn and Fayoumi even under normal non-challenged conditions. With this result, we can deduce the differences between both breeds in lung transcriptome response, similar to that identified in our previous studies on the trachea and harderian gland. However, the number of DEGs and DElncRNAs was lower in the case of the lung transcriptome.

In functional annotation of genes, it was found that a total of 25 different genes were annotated with the biological process Immune system process (GO:0002376), and 18 different genes were annotated with the biological process response to biotic stimulus (GO:0009607). A total of 45 different genes were annotated with pathways in the Immune system (Reactome) category, which were annotated with different biological process GOs, including biological regulation (GO:0065007), cellular process (GO:0009987), immune system process (GO:0002376), response to biotic stimulus (GO:0009607) and Signalling (GO:0023052). Overall, 66 genes were annotated with either immune-related biological process GOs or pathways. Of this, 12 genes were annotated with both biological process GOs and Immune system category pathways. In challenge and timepoint-based analysis, only one gene, MSTRG.18444, was found to be differentially expressed. It was found to be downregulated in challenge-based analysis in Fayoumi at 6DPC and upregulated in timepoint-based analysis in Fayoumi 6 vs 10 DPC. In breed-based analysis, the genes IL6ST, MSTRG.10718, and MSTRG.4083 were upregulated in Fayoumi and the genes MSTRG.12549 and MSTRG.18444 were found to be downregulated in Fayoumi than in Leghorn in breed-based analysis during both challenged and non-challenged conditions. The other 7 genes, i.e., CTSS, MSTRG.14170, MSTRG.14920, MSTRG.5632, MSTRG.8108, MSTRG.9061 and NPC2 were upregulated in Fayoumi at 10 DPC in non-challenged samples. Apart from this, of the 66 genes annotated with either immune GO or immune pathway, several genes were upregulated in challenge-based analysis in Fayoumi but not in Leghorn. A few genes were also downregulated between time points in timepoint-based analysis in Leghorn but not in Fayoumi. This pattern shows the differences in the breeds even in the absence of any infection. In addition to this, several non-immune genes involved in pathways, including metabolism, signal transduction, transport of small molecules, extracellular matrix organisation, developmental biology and cellular processes, were impacted by NDV infection.

In GO functional enrichment analysis, several enriched GOs and pathways were identified. No enriched immune-related GOs were identified across all the datasets. In breed-based analysis, only one enriched immune pathway was identified in non-challenge 10 DPC data. In Leghorn challenge vs non-challenge data, enriched biological process GOs were identified only at Leghorn 6 DPC. The highest number of enriched GOs were found under the developmental process followed by biological regulation. All the enriched GOs were found to have positive NES, indicating upregulated GOs. In the Fayoumi challenge vs non-challenge data, enriched biological process GOs were identified at 2, 6 and 10 DPC. At 2 DPC, metabolic process and cellular process GOs were enriched with positive NES. At 6 DPC, enriched GOs were found under biological regulation and developmental process, with negative NES indicating downregulation. In comparison, at 10 DPC, biological regulation and cellular process GOs were enriched with positive NES, indicating upregulation. In breed-based analysis, non-challenge data showed enriched GOs at 6 DPC under metabolic process and at 10 DPC under biological regulation followed by metabolic and developmental process with positive NES. Challenge data showed enriched GOs at 2, 6 and 10 DPC. The most enriched GOs were found under biological regulation, followed by the developmental process with positive NES at all three timepoints. There were no enriched biological process GOs in timepoint-based analysis. In the case of pathway enrichment analysis, no enriched pathways were identified in the challenge-based analysis. In breed-based analysis, all six conditions showed enriched pathways, most belonging to the Disease (Reactome) category with negative NES. A few metabolic and immune system pathways were identified in non-challenged samples with positive NES. In timepoint-based analysis, only Fayoumi 2 vs 10 DPC data showed two enriched pathways under the transport of small molecules category with negative NES. These results show the impact of NDV on various non-immune pathways, unlike previous analyses on the trachea and harderian gland, in which several enriched immune-related GOs and pathways were identified.

In co-expression analysis between the DEGs and DElncRNAs, it was observed that most of the co-expression pairs were trans than cis. As mentioned earlier, cis co-expression lncRNAs were considered to regulate the genes near them. In challenge-based analysis, only Leghorn 6 DPC and Fayoumi 10 DPC have shown cis co-expression pairs. Leghorn showed only 1 pair with the gene involved in sensory perception. In Fayoumi, cis-pairs had genes involved in various pathways under the immune system, metabolic, cellular process, signal transduction, DNA repair, response to stimulus and developmental biology. This result clearly shows the differences in the lncRNA transcriptome response between the Leghorn and Fayoumi breeds during the NDV challenge. In breed-based analysis, both challenged and non-challenged datasets have shown several gene-lncRNAs pairs with genes involved in various pathways under metabolism, immune system, disease, signal transduction, metabolic and cellular processes, transport of molecules and developmental pathways. In timepoint-based analysis, only Leghorn have shown cis co-expression pairs with genes involved in sensory perception and biological regulatory pathways. This analysis shows that the lncRNAs might have a potential role in differentiating these breeds. A higher number of the pairs were shown to have a positive correlation, indicating that most of the DElncRNAs could positively regulate the co-expressing genes. However, several negatively regulating DElncRNAs were also identified, even with immune-related genes.

In addition, several transcription factors and microRNAs were identified to be interacting with the DEGs. A total of 10 immune annotated genes were identified to be interacting with transcription factors, of which six genes showed three all motifs (1, 4, 6) and 34 TFs, two genes showed two motifs (4, 6) and 33 TFs, and two genes showed one each motif – motif 4 and 12 TFs, and motif 6 and 32 TFs respectively). Similar to the previous reports on the trachea and harderian gland, TFs from three families – E2F, ZBTB (Zinc finger and BTB domain containing) and zf-C2H2 (zinc finger Cys2–His2) were identified. These were known to regulate apoptotic (Xanthoulis and Tiniakos, 2013), T-lymphocyte-related molecular mechanisms (Cheng et al., 2021) and disease developmental processes (Han et al., 2016), respectively. Only two immune genes were found to be targeted by 14 microRNAs. – gene CTSS (3) and NPC2 (12). Along with co-expressing cis-lncRNAs, this information on TFs and miRNAs can be used to regulate the expression of immune genes in further studies. QTL analysis has shown that most of the DEGs identified in different conditions were mapped to production and health QTLs. Breed-based QTL analysis showed the highest number of genes mapped to QTLs of the three methods. In challenge-based analysis, Fayoumi showed the highest number of genes with overall QTLs and the highest number of health QTLs compared to Leghorn. However, in timepoint-based analysis, Leghorn showed a higher number of Health QTLs than Fayoumi, which could mean that Leghorn shows more differential expression during the disease than Fayoumi, which is comparatively resistant and could have fewer differences in expression between different timepoints of the disease. The breed-based analysis has shown the health QTLs in both challenged and non-challenged datasets, indicating the difference between Leghorn and Fayoumi, even in the normal state.

Overall, in this analysis, most of the 12 genes annotated with immune-related GOs and pathways were upregulated in Fayoumi, compared to Leghorn during the NDV challenge. Such immune gene upregulation was even observed during non-challenged conditions. In addition, in challenge-based analysis, NDV-challenged Fayoumi showed a higher number of upregulated immune genes compared to non-challenged Fayoumi, while there was no such upregulation in Leghorn. Similarly, a higher number of cis-lncRNAs were identified in Fayoumi compared to Leghorn. Most of these cis-lncRNAs were positively correlated with the immune-related genes. Previous studies by Melissa et al. show that the presence of viral transcripts in the tissue significantly helps in eliciting immune response and upregulation of immune-related genes, which was also observed in our earlier work on the trachea and harderian gland transcriptome analysis, where higher levels are viral transcripts were observed in those tissues. Unlike those tissues, lung tissue from both Leghorn and Fayoumi showed no viral transcripts. With this, we can understand that Fayoumi chicken showed upregulated immune genes and positive cis-lncRNAs during both the non-challenged and NDV-challenge conditions, even without viral transcripts in the tissue. This finding shows that these immune-annotated genes and co-expressing cis-lncRNAs play a significant role in Fayoumi being comparatively resistant to NDV compared to Leghorn. Our study affirms and expands upon the outcomes of previous studies and highlights the crucial role of lncRNAs during the immune response to NDV. Although this analysis is robust, certain limitations exist. This study is limited to in-silico analysis of transcriptome data obtained from lung tissue and experimental validation of a few lncRNAs and co-expressing genes. To better understand the overall response of the host and identify the role of immune genes and lncRNAs, the analysis of different tissues during NDV is required. Future studies can unravel the mechanisms of co-expression and interaction of DEGs and DElncRNAs. This information about these twelve immune-annotated genes and lncRNAs co-expressing with them can be used to improve resistance in susceptible breeds.

## Conclusion

The lung is one of the essential organs in chickens and plays a vital role in the immune response against the Newcastle disease virus. In this analysis of the lung transcriptome, most of the immune-related genes were upregulated in Fayoumi in challenged and non-challenged conditions compared to Leghorn. In addition, several immune genes were upregulated in Fayoumi non-challenge vs challenge but not in Leghorn. Several gene ontologies under developmental process and biological regulation categories were enriched with positive NES (upregulated) in Fayoumi but not in Leghorn. In the case of pathways, few immune system and metabolic pathways were found to be enriched with positive NES (upregulated) between Leghorn and Fayoumi non-challenge data, indicating the difference between the breeds. A similar trend was observed in the case of lncRNAs. Several co-expressing pairs included lncRNAs positively correlated with both immune and non-immune genes. Fayoumi showed several positively correlated cis-lncRNAs co-expressing with immune-related genes, while no such lncRNAs were identified in Leghorn. This analysis clearly shows the differences in the gene expression patterns and lncRNA co-expression with the genes between Leghorn and Fayoumi, indicating that the lncRNAs and co-expressing genes might potentially have a role in differentiating these breeds. This study shows the transcriptomic response. An in-depth protein level analysis will help understand the exact mechanism in regulating genes and the role of the lncRNAs in regulating these genes.

## Data Availability

Publicly available datasets were analysed in this study. This data can be found here: EBI-ENA database with project ID PRJEB21760. The pipeline used in the study – FHSpipe can be found here: https://github.com/Venky2804/FHSpipe.

## Footnotes

1 GraphPad Software. Multiple unpaired t-tests were performed by using GraphPad Prism v9.4.1 for WIndows (San Diego, California, USA).

## Supplementary material

Supplementary Figure 1 Figure showing the heatmap of the read counts of differentially expressed genes.

Supplementary Figure 2 Figure showing the heatmap of the read counts of differentially expressed lncRNAs.

## References

[B1] Altschul S. Gish W. Miller W. Myers E. Lipman D. (1990). Basic local alignment search tool. J. Mol. Biol. 215, 403–410. doi: 10.1016/S0022-2836(05)80360-2 2231712

[B2] Bailey T. L. Boden M. Buske F. A. Frith M. Grant C. E. Clementi L.. (2009). MEME SUITE: tools for motif discovery and searching. Nucleic Acids Res. 37, W202–W208. doi: 10.1093/nar/gkp335 19458158 PMC2703892

[B3] BioBam Bioinformatics (2019). OmicsBox - Bioinformatics made easy.

[B4] Chan P. P. Lowe T. M. (2009). GtRNAdb: A database of transfer RNA genes detected in genomic sequence. Nucleic Acids Res. 37, D93–D97. doi: 10.1093/nar/gkn787 18984615 PMC2686519

[B5] Chang L. Zhou G. Soufan O. Xia J. (2020). miRNet 2.0: network-based visual analytics for miRNA functional analysis and systems biology. Nucleic Acids Res. 48, (W1) W244–W251. doi: 10.1093/nar/gkaa467 32484539 PMC7319552

[B6] Cheeseman J. H. Kaiser M. G. Ciraci C. Kaiser P. Lamont S. J. (2007). Breed effect on early cytokine mRNA expression in spleen and cecum of chickens with and without Salmonella enteritidis infection. Dev. Comp. Immunol. 31, 52–60. doi: 10.1016/j.dci.2006.04.001 16762413

[B7] Chen S. Zhou Y. Chen Y. Gu J. (2018). fastp: an ultra-fast all-in-one FASTQ preprocessor. Bioinformatics (Oxford, England) 34 (17), i884–i890. doi: 10.1093/bioinformatics/bty560 30423086 PMC6129281

[B8] Cheng Z. Y. He T. T. Gao X. M. Zhao Y. Wang J. (2021). ZBTB transcription factors: key regulators of the development, differentiation and effector function of T cells. Front. Immunol. 12, 713294. doi: 10.3389/fimmu.2021.713294 34349770 PMC8326903

[B9] Deist M. S. Gallardo R. A. Bunn D. A. Dekkers J. Zhou H. Lamont S. J. (2017). Resistant and susceptible chicken lines show distinctive responses to Newcastle disease virus infection in the lung transcriptome. BMC Genomics 18, 989. doi: 10.1186/s12864-017-4380-4 29281979 PMC5745900

[B10] Dimitrov K. M. Afonso C. L. Yu Q. Miller P. J. (2017). Newcastle disease vaccines-A solved problem or a continuous challenge? Vet. Microbiol. 206, 126–136. doi: 10.1016/j.vetmic.2016.12.019 28024856 PMC7131810

[B11] Fabregat A. Jupe S. Matthews L. Sidiropoulos K. Gillespie M. Garapati P.. (2018). The reactome pathway knowledgebase. Nucleic Acids Res. 46, D649–D655. doi: 10.1093/nar/gkx1132 29145629 PMC5753187

[B12] Ganar K. Das M. Sinha S. Kumar S. (2014). Newcastle disease virus: current status and our understanding. Virus Res. 184, 71–81. doi: 10.1016/j.virusres.2014.02.016 24589707 PMC7127793

[B13] Gotz S. Garcia-Gomez J. Terol J. Williams T. Nagaraj S. Nueda M.. (2008). High-throughput functional annotation and data mining with the Blast2GO suite. Nucleic Acids Res. 36, 3420–3435. doi: 10.1093/nar/gkn176 18445632 PMC2425479

[B14] Guttman M. Rinn J. (2012). Modular regulatory principles of large non-coding RNAs. Nature 482, 339–346. doi: 10.1038/nature10887 22337053 PMC4197003

[B15] Han B. Y. Foo C. S. Wu S. Cyster J. G. (2016). The C2H2-ZF transcription factor Zfp335 recognizes two consensus motifs using separate zinc finger arrays. Genes Dev. 30, 1509–1514. doi: 10.1101/gad.279406.116 27401554 PMC4949324

[B16] Hassan M. K. Afify M. A. Aly M. M. (2004). Genetic resistance of Egyptian chickens to infectious bursal disease and Newcastle disease. Trop. Anim. Health production 36, 1–9. doi: 10.1023/B:TROP.0000009524.47913.d4 14979553

[B17] Huerta-Cepas J. Szklarczyk D. Heller D. Hernández-Plaza A. Forslund S. K. Cook H.. (2019). eggNOG 5.0: a hierarchical, functionally and phylogenetically annotated orthology resource based on 5090 organisms and 2502 viruses. Nucleic Acids Res. 47, (D1) D309–D314. doi: 10.1093/nar/gky1085 30418610 PMC6324079

[B18] Hwang J. Y. Randall T. D. Silva-Sanchez A. (2016). Inducible bronchus-associated lymphoid tissue: taming inflammation in the lung. Front. Immunol. 7, 258. doi: 10.3389/fimmu.2016.00258 27446088 PMC4928648

[B19] Kanehisa M. Goto S. (2000). KEGG: Kyoto Encyclopedia of genes and genomes. Nucleic Acids Res. 28, 27–30. doi: 10.1093/nar/28.1.27 10592173 PMC102409

[B20] Kang Y. J. Yang D. C. Kong L. Hou M. Meng Y. Q. Wei L.. (2017). CPC2: a fast and accurate coding potential calculator based on sequence intrinsic features. Nucleic Acids Res. 45, W12–W16. doi: 10.1093/nar/gkx428 28521017 PMC5793834

[B21] Kim D. Paggi J. M. Park C. Bennett C. Salzberg S. L. (2019). Graph-based genome alignment and genotyping with HISAT2 and HISAT-genotype. Nat. Biotechnol. 37, 907–915. doi: 10.1038/s41587-019-0201-4 31375807 PMC7605509

[B22] Kozomara A. Birgaoanu M. Griffiths-Jones S. (2019). MiRBase: From microRNA sequences to function. Nucleic Acids Res. 47, D155–D162. doi: 10.1093/nar/gky1141 30423142 PMC6323917

[B23] Krzywinski M. Schein J. Birol I. Connors J. Gascoyne R. Horsman D.. (2009). Circos: an information aesthetic for comparative genomics. Genome Res. 19, 1639–1645. doi: 10.1101/gr.092759.109 19541911 PMC2752132

[B24] Langfelder P. Horvath S. (2008). WGCNA: an R package for weighted correlation network analysis. BMC Bioinf. 9, 559. doi: 10.1186/1471-2105-9-559 PMC2631488 19114008

[B25] Li J. Li R. Wang Y. Hu X. Zhao Y. Li L.. (2015). Genome-wide DNA methylome variation in two genetically distinct chicken lines using MethylC-seq. BMC Genomics 16, 851. doi: 10.1186/s12864-015-2098-8 26497311 PMC4619007

[B26] Mahmoud S E.-T. (2019). Impact of crossing Fayoumi and Leghorn chicken breeds on immune response against Newcastle disease virus vaccines. Trop. Anim. Health Prod. 51, 429–434. doi: 10.1007/s11250-018-1709-1 30219997

[B27] Mebrate G. Tewodros A. Dawit A. Daniel G. Derbie Z. (2019). Epidemiology, diagnosis & Prevention of newcastle disease in poultry. Am. J. Biomed. Sci. Res. 3, 50–59. doi: 10.34297/AJBSR

[B28] Pertea G. Pertea M. (2020). GFF utilities: gffRead and gffCompare. F1000Research 9, J–304. doi: 10.12688/f1000research PMC7222033 32489650

[B29] Pertea M. Pertea G. M. Antonescu C. M. Chang T. C. Mendell J. T. Salzberg S. L. (2015). StringTie enables improved reconstruction of a transcriptome from RNA-seq reads. Nat. Biotechnol. 33, 290–295. doi: 10.1038/nbt.3122 25690850 PMC4643835

[B30] Philip J. David B. Hsin-Yu C. Matthew F. Weizhong L. Craig M.. (2014). InterProScan 5: genome-scale protein function classification. Bioinformatics 30, 1236–1240. doi: 10.1093/bioinformatics/btu031 24451626 PMC3998142

[B31] Pinard-van der Laan M. H. Bed'hom B. Coville J. L. Pitel F. Feve K. Leroux S.. (2009). Microsatellite mapping of QTLs affecting resistance to coccidiosis (Eimeria tenella) in a Fayoumi x White Leghorn cross. BMC Genomics 10, 31. doi: 10.1186/1471-2164-10-31 19154572 PMC2633352

[B32] Quast C. Pruesse E. Yilmaz P. Gerken J. Schweer T. Yarza P.. (2013). The SILVA ribosomal RNA gene database project: improved data processing and web-based tools. Nucleic Acids Res. 41, D590–D596. doi: 10.1093/nar/gks1219 23193283 PMC3531112

[B33] Robinson M. D. McCarthy D. J. Smyth G. K. (2010). edgeR: a Bioconductor package for differential expression analysis of digital gene expression data. Bioinformatics 26, 139–140. doi: 10.1093/bioinformatics/btp616 19910308 PMC2796818

[B34] Schilling M. A. Memari S. Cavanaugh M. Katani R. Deist M. S. Radzio-Basu J.. (2019). Conserved, breed-dependent, and subline-dependent innate immune responses of Fayoumi and Leghorn chicken embryos to Newcastle disease virus infection. Sci. Rep. 9, 7209. doi: 10.1038/s41598-019-43483-1 31076577 PMC6510893

[B35] Shannon P. Markiel A. Ozier O. Baliga N. S. Wang J. T. Ramage D.. (2003). Cytoscape: a software environment for integrated models of biomolecular interaction networks. Genome Res. 13, 2498–2504. doi: 10.1101/gr.1239303 14597658 PMC403769

[B36] Shen W. K. Chen S. Y. Gan Z. Q. Zhang Y. Z. Yue T. Chen M. M.. (2023). AnimalTFDB 4.0: a comprehensive animal transcription factor database updated with variation and expression annotations. Nucleic Acids Res. 51, (D1) D39–D45. doi: 10.1093/nar/gkac907 36268869 PMC9825474

[B37] Subramanian A. Tamayo P. Mootha V. K. Mukherjee S. Ebert B. L. Gillette M. A.. (2005). Gene set enrichment analysis: a knowledge-based approach for interpreting genome-wide expression profiles. Proc. Natl. Acad. Sci. U.S.A 102, 15545–15550. doi: 10.1073/pnas.0506580102 16199517 PMC1239896

[B38] Vanamamalai V. K. Garg P. Kolluri G. Gandham R. K. Jali I. Sharma S. (2021). Transcriptomic analysis to infer key molecular players involved during host response to NDV challenge in Gallus gallus (Leghorn & Fayoumi). Sci. Rep. 11(1), 8486. doi: 10.1038/s41598-021-88029-6 33875770 PMC8055681

[B39] Vanamamalai V. K. Priyanka E. Kannaki T. R. Sharma S. (2023). Integrated analysis of genes and long non-coding RNAs in trachea transcriptome to decipher the host response during Newcastle disease challenge in different breeds of chicken. Int. J. Biol. Macromol. 253, (Pt 5) 127183. doi: 10.1016/j.ijbiomac.2023.127183 37793531

[B40] Venkata Krishna V. Priyanka E. Kannaki T. R. Shailesh S. (2024). Breed and timepoint-based analysis of chicken harderian gland transcriptome during Newcastle Disease Virus challenge. Front. Mol. Biosci. 11, 1365888. doi: 10.3389/fmolb.2024.1365888 38915939 PMC11194529

[B41] Wang Y. Lupiani B. Reddy S. M. Lamont S. J. Zhou H. (2014). RNA-seq analysis revealed novel genes and signaling pathway associated with disease resistance to avian influenza virus infection in chickens. Poultry Sci. 93, 485–493. doi: 10.3382/ps.2013-03557 24570473

[B42] Warnes G. Bolker B. Bonebakker L. Gentleman R. Huber W. Liaw A.. (2020). gplots: Various R Programming Tools for Plotting Data.

[B43] Xanthoulis A. Tiniakos D. G. (2013). E2F transcription factors and digestive system Malignancies: how much do we know? World J. Gastroenterol. 19, 3189–3198. doi: 10.3748/wjg.v19.i21.3189 23745020 PMC3671070

[B44] Ye J. Coulouris G. Zaretskaya I. Cutcutache I. Rozen S. Madden T. L. (2012). Primer-BLAST: a tool to design target-specific primers for polymerase chain reaction. BMC Bioinf. 13, 134. doi: 10.1186/1471-2105-13-134 PMC3412702 22708584

[B45] Zhao L. Wang J. Li Y. Song T. Wu Y. Fang S.. (2021). NONCODEV6: an updated database dedicated to long non-coding RNA annotation in both animals and plants. Nucleic Acids Res. 49, D165–D171. doi: 10.1093/nar/gkaa1046 33196801 PMC7779048

[B46] Zhao R. Li J. Liu N. Li H. Liu L. Yang F.. (2020). Transcriptomic analysis reveals the involvement of lncRNA–miRNA–mRNA networks in hair follicle induction in Aohan fine wool sheep skin. Front. Genet. 11, 590. doi: 10.3389/fgene.2020.00590 33117415 PMC7528302

