# Supplementary figures and images for: Integrative study of chicken lung transcriptome to understand the host immune response during Newcastle disease virus challenge
Source: Front Cell Infect Microbiol. 2024 Sep 3;14:1368887. doi: 10.3389/fcimb.2024.1368887 (PMC11405381; doi:10.3389/fcimb.2024.1368887)

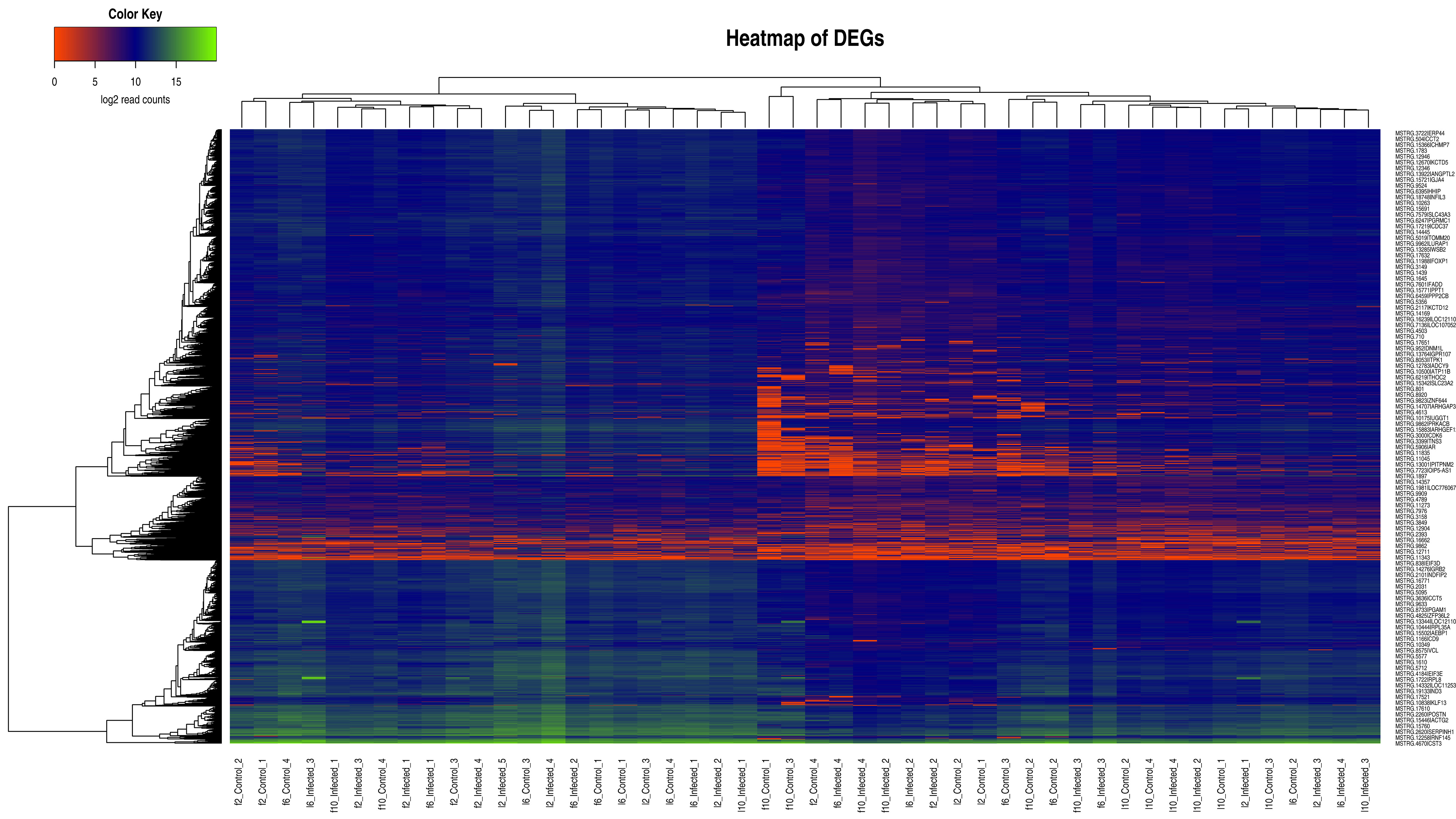

Supplement: Supplementary Figure 1 — Figure showing the heatmap of the read counts of differentially expressed genes. [file Image1.jpeg]

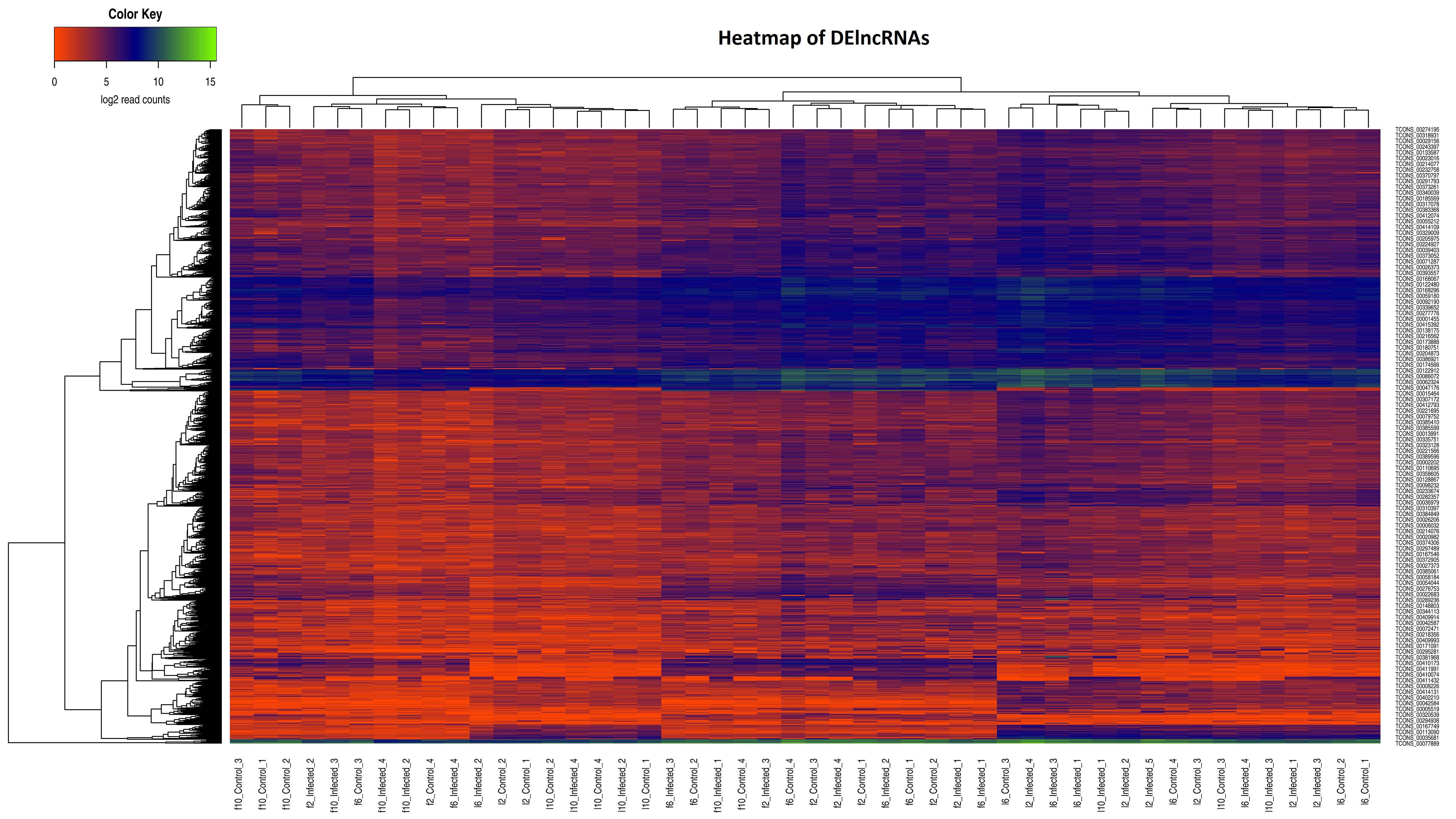

Supplement: Supplementary Figure 2 — Figure showing the heatmap of the read counts of differentially expressed lncRNAs. [file Image2.jpeg]
